# Estimating the contribution of studies in network meta-analysis: paths, flows and streams

**DOI:** 10.12688/f1000research.14770.3

**Published:** 2018-12-13

**Authors:** Theodoros Papakonstantinou, Adriani Nikolakopoulou, Gerta Rücker, Anna Chaimani, Guido Schwarzer, Matthias Egger, Georgia Salanti

**Affiliations:** 1 Institute of Social and Preventive Medicine (ISPM), University of Bern, Bern, Switzerland; 2 Institute of Medical Biometry and Statistics, Faculty of Medicine and Medical Center, University of Freiburg, Freiburg, Germany; 3 Paris Descartes University, INSERM, UMR1153 Epidemiology and Statistics, Sorbonne Paris Cité Research Center (CRESS), METHODS Team; Cochrane France, Paris, France

**Keywords:** indirect evidence, proportion contributions, projection matrix, flow networks

## Abstract

In network meta-analysis, it is important to assess the influence of the limitations or other characteristics of individual studies on the estimates obtained from the network. The proportion contribution matrix, which shows how much each direct treatment effect contributes to each treatment effect estimate from network meta-analysis, is crucial in this context. We use ideas from graph theory to derive the proportion that is contributed by each direct treatment effect. We start with the ‘projection’ matrix in a two-step network meta-analysis model, called the ***H ***matrix, which is analogous to the hat matrix in a linear regression model. We develop a method to translate ***H ***entries to proportion contributions based on the observation that the rows of ***H*** can be interpreted as flow networks, where a stream is defined as the composition of a path and its associated flow. We present an algorithm that identifies the flow of evidence in each path and decomposes it into direct comparisons. To illustrate the methodology, we use two published networks of interventions. The first compares no treatment, quinolone antibiotics, non-quinolone antibiotics and antiseptics for underlying eardrum perforations and the second compares 14 antimanic drugs. We believe that this approach is a useful and novel addition to network meta-analysis methodology, which allows the consistent derivation of the proportion contributions of direct evidence from individual studies to network treatment effects.

## Introduction

Decision making around multiple alternative healthcare interventions is increasingly based on meta-analyses of a network of relevant studies, which contribute direct and indirect evidence to different treatment comparisons ^1, 2^. Limitations in the design and flaws in the conduct of studies synthesized in network meta-analysis (NMA) reduce the confidence in the results: a treatment comparison in the network may be directly or indirectly informed by studies at high risk of bias. A relative treatment effect from NMA (hereafter the NMA effect estimate) is estimated as a linear combination of the available direct estimates of the treatment effect (i.e. the results from pairwise meta-analyses) and the indirect evidence on the treatment effect.

Salanti *et al*. suggested that in order to assess the impact of study deficiencies on an NMA effect estimate, the limitations of studies contributing to direct estimates should be considered jointly, taking into account their relative contribution to the overall NMA effect estimate ^3^. The proportion contribution matrix plays a key role in this approach: a matrix that shows how much each direct effect contributes to the estimation of the NMA effect.

The proportion contribution matrix is derived from the absolute contribution matrix. The absolute contributions of direct effects to an NMA effect is the projection matrix from a two-step NMA model ^4, 5^. In the first stage, all direct effects are derived from pairwise meta-analyses. In the second stage, the NMA effect estimates are produced as a linear combination of the derived direct effects. The respective projection matrix is called the ***H*** matrix and it is analogous to the hat matrix in a linear regression model. The elements in the ***H*** matrix can be viewed as generalized weights from pairwise meta-analysis, but they do not add up to 1 and depend on the precision of the available studies, the degree of between-study heterogeneity and the network structure.

To translate the entries of the ***H*** matrix into proportion contributions, Salanti *et al*. suggested normalizing the absolute entries of each row of ***H*** and interpret them as proportions ^3^. However, ***H*** represents the flow of evidence in different paths; the weight of each path is assigned to each direct effect involved. Thus, ignoring the multiple occurrences of the same values by taking standardized absolute values is incorrect. In particular, such a process overestimates the contribution of comparisons involved in long paths and underestimates the weights of the shortest paths. In this paper, we address this issue and present a method that properly translates the entries of the ***H*** matrix into proportions. The methodology is based on the observation that the rows of the ***H*** matrix can be interpreted as flow networks ^4, 6^.

### Motivating example

**Figure 1.**
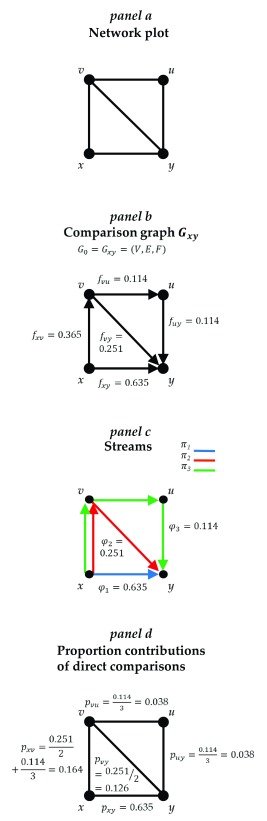
Network plot for the network of topical antibiotics without steroids for chronically discharging ears (a), comparison graph corresponding to the *h ^xy^* row of *H* matrix (b), flows *f _uv_* with respect to the ‘ *x* versus *y*’ network meta-analysis treatment effect are indicated along the edges), streams (c) and proportion contributions of each direct comparison (d). ***x***, no treatment; ***y***, quinolone antibiotic; ***u***, non-quinolone antibiotic; ***v***, antiseptic.

To illustrate the ideas presented in this paper, we will use a network of topical antibiotics for the treatment of chronic otitis media with ear discharge in patients with eardrum perforations ^7^. This network was used in Salanti *et al.* ^3^ and compares no treatment ( *x*), quinolone antibiotic treatment ( *y*), non-quinolone antibiotic treatment ( *u*) and antiseptic treatment ( *v*) ^7^. The study outcome was the proportion of patients with persistent discharge from the ear after 1 week, measured using the odds ratio (OR). The network plot shown in Figure 1a shows that direct evidence exists for all comparisons except *u* versus *x* (non-quinolone antibiotic versus no treatment).

In order to assess the confidence that should be placed in an NMA effect estimate, Salanti *et al*. suggested considering the quality of all pieces of evidence that contributed to it ^3^. For example, the studies directly comparing ‘ *u* versus *v*’ were judged to be at high risk of bias; however, in order to judge the quality of the NMA effect estimate of ‘ *u* versus *v*’, we need to consider the amount of data that these studies contributed to its estimation.

## Methods

We first present the random-effects two-stage NMA model first described by Lu *et al*. ^5^. We will employ a simplified version of the ***H*** matrix described by König *et al.* ^4^ that does not take into account the correlation induced by multi-arm trials. We ignore this correlation for the sake of ease of interpretation of the entries in the ***H*** matrix; we discuss implications of multi-arm trials at the end of the Methods section. Taking advantage of previous findings on how the flow of evidence can be considered in NMA ^4, 6^, we present an algorithm to decompose the flow in a network and subsequently approximate the proportion contributions of direct effect estimates for each NMA effect estimate.

### Two-stage network meta-analysis model

Consider a network of *T* competing treatments. The set of treatments is denoted by *V* = { *x*, *y*, *u*, *v*, ...} and let *x* denote the reference treatment. The number of NMA effects to be estimated is $\left(\begin{array}{l} T\\ 2 \end{array}\right)$ but the estimation of *T* – 1 effects allows the derivation of the remaining effects via linear combination. We collect the *T* – 1 effects against the reference treatment *x* in a vector of basic parameters ***θ*** = ( *θ _xy_,θ _xu_,θ _xv_, ...*)′. In the case of a dichotomous outcome, ***θ*** is the parameter vector of all log-ORs compared to the common reference treatment *x*.

We assume that the distribution of effect modifiers is similar across comparisons and thus the transitivity assumption is plausible. The consistency assumption refers to the statistical manifestation of transitivity and implies that all sources of evidence are in agreement; this is expressed via the consistency equations

*θ _uv_* = *θ _xv_* – *θ _xu_*, for all *u*, *v* ∈ *V*

Let us denote the number of comparisons with direct data (that is, at least one direct study) with *D*. For simplification, consider that there are no multi-arm studies. At the first stage of the NMA model, direct effects are estimated, using random-effects pairwise meta-analyses. The estimates of the direct effects are collected in a column vector ${\hat{\theta }}^{D}$ of length *D*; their estimated variances are collected in a diagonal *D* × *D* matrix ***V ^D^***. At the second stage, the NMA effects are estimated as

$${\hat{\theta }}^{N}=H{\hat{\theta }}^{D}\;\mathrm{Equation}1$$

where ***H*** is

***H = Y*( *X′*( *V ^D^*) ^–1^*X*) ^–1^*X′*( *V ^D^*) ^–1^** **Equation   2**

Matrix ***X*** is a *D* × ( *T* – 1) design matrix expressing the linear relationships between the available direct effects and the basic parameters and ***Y*** is a $\left(\begin{array}{l} T\\ 2 \end{array}\right)$ × ( *T* – 1) design matrix that links the NMA estimates with the basic parameters. Note that ***X*** is identical to ***Y*** only when there are direct studies for all treatment comparisons in the network.

Matrix ***H*** is of dimensions $\left(\begin{array}{l} T\\ 2 \end{array}\right)$ × *D* and describes the influence of each direct effect (specified in the column) to an NMA effect (specified in the row). As Equation 2 implies, ***H*** is derived as a function of the variances of the direct effects ${\hat{\theta }}^{D}$ and the network structure; therefore, the exact (absolute) contribution of each direct comparison depends on the precision of the available direct data and the comparison’s connectivity to the rest of the network. Note that it resembles the hat matrix in a linear regression model.

Let us focus on a single row of the ***H*** matrix which, say, corresponds to the NMA effect estimate of ‘ *x* versus *y*’ and is denoted by ***h ^xy^***. Elements of ***h ^xy^*** are denoted by $h_{uv}^{xy}$ and show the *absolute* contribution of the direct effect ${\hat{\theta }}_{uv}^{D}$ indicated in the subscript (‘ *u* versus *v*’) to the ‘ *x* versus *y*’ NMA effect ${\hat{\theta }}_{xy}^{N}$. Consider our motivating example which examines the set of treatments *V* = { *x*, *y*, *u*, *v*}. Equation 1 implies that the NMA treatment effect for the ‘ *x* versus *y*’ comparison is derived as a linear combination of the direct meta-analyses

$${\hat{\theta }}_{xy}^{N}=h_{xy}^{xy}{\hat{\theta }}_{xy}^{D}+h_{yv}^{xy}{\hat{\theta }}_{yv}^{D}+h_{xv}^{xy}{\hat{\theta }}_{xv}^{D}+h_{yu}^{xy}{\hat{\theta }}_{yu}^{D}+h_{uv}^{xy}{\hat{\theta }}_{uv}^{D}\;\mathrm{Equation}3$$

The element $h_{xy}^{xy}$ represents the absolute but also the *proportion* contribution $p_{xy}^{xy}$ of the direct evidence for the particular NMA effect. Assuming that the comparison ‘ *x* versus *y*’ is not part of any multi-arm study, the evidence to derive the NMA effect estimate can be portioned into direct and indirect estimates

$${\hat{\theta }}_{xy}^{N}=h_{xy}^{xy}{\hat{\theta }}_{xy}^{D}+(1-h_{xy}^{xy}){\hat{\theta }}_{xy}^{I}\;\mathrm{Equation}4$$

with ${\hat{\theta }}_{xy}^{I}$ denoting the indirect effect for the ‘ *x* versus *y*’ comparison; hence $p_{xy}^{xy}=h_{xy}^{xy}$. While the proportion contribution of each direct effect to its NMA effect can be obtained as the diagonal of the ***H*** matrix, the proportion contributions of other direct relative effects via indirect evidence (e.g. the $p_{uv}^{xy}$ proportion contribution of ${\hat{\theta }}_{uv}^{D}$ to ${\hat{\theta }}_{xy}^{N}$) cannot be easily derived from the absolute contributions (that is, from $h_{uv}^{xy}$). In the next section we will present how the absolute contributions $h_{uv}^{xy}$ could be translated to proportion contributions $p_{uv}^{xy}$.

To explain the method, we will continue focusing on one row of the ***H*** matrix, say ***h ^xy^***, corresponding to the ‘ *x* versus *y*’ comparison. Box 1 includes the definitions, along with the notation, of some of the notions used in this paper.

Box 1. **Definitions**

**Set of vertices**

The set of vertices is defined as the set of treatments examined in the network, *V* = { *x, y, u, v ...*}.

**Set of directed edges**

The set of directed edges *E* is defined as the set of direct comparisons respecting the signs of the entries of ***h ^xy^***, the row of the ***H*** matrix corresponding to the ‘ *x* versus *y*’ comparison. Edges are given a direction upon the definition of flows; the network itself corresponds to an undirected graph.

**Set of flows**

The set of flows is defined as *F* = { *f _uv_, ∀ uv ∈ E*} where *f _uv_* is equal to | *h _uv_*|.

**Comparison graph**

A comparison graph is defined as a graph *G _xy_* = ( *V, E, F*) constructed from a row of the ***H*** matrix, ***h ^xy^***; its definition derives from a set of vertices *V*, a set of edges *E*, and a set of flows, *F*.

**Source**

Source is defined as a vertex with no incoming edges.

**Sink**

Sink is defined as a vertex with no outgoing edges.

**Path**

A path *π _i_* is defined as a sequence of connected directed edges belonging to *E*.

**Stream**

A stream *S _i_* is defined as the composition of a path and its associated flow, *S _i_* = ( *φ _i_*, *π _i_*). with *i* = 1, ... , *I* where *I* is the total number of streams.

### Comparison graph

König *et al*. showed that every row of the ***H*** matrix, ***h ^xy^***, can be interpreted as a flow network with source *x* and sink *y*, and visualised in a directed acyclic graph (DAG) ^4^. Thus, we create a graph *G _xy_* = ( *V, E, F*) from ***h ^xy^***; its definition derives from a set of vertices *V*, a set of edges *E*, and a set of flows, *F*. The set of vertices is defined as the set of treatments examined in the network, *V* = { *x*, *y*, *u*, *v* ...}. Set *E* is defined as a set of directed edges that correspond to observed direct comparisons respecting the signs of the entries of ***h ^xy^***. To simplify the notation, we drop from now on all superscripts assuming they all refer to *xy*. Then, the set *E* contains *uv* if *h _uv_* > 0 or contains *vu* if *h _uv_* < 0. The set of flows is defined as *F* = { *f _uv_*, ∀ *uv* ∈ *E*} where *f _uv_* is equal to | *h _uv_*|.

The following conditions hold for the elements of set *F* (see Supplementary File 1 for proof):

      a. The sum of outflows of node *x* (source) is 1

$$\sum_{xu\in E}f_{xu}=1$$

      b. The sum of inflows of node *y* (sink) is 1

$$\sum_{uy\in E}f_{uy}=1$$

      c. The flow passing through each internal node (any node except *x or y*) is conserved

$$\forall z\in V\backslash \{x,y\},\sum_{v\in V}f_{vz}=\sum_{u\in V}f_{zu}$$

      d. *G _xy_* is acyclic; there is no path (sequence of edges) that visits the same vertex twice.

Consider, for example, the graph *G _xy_* in Figure 1b, which corresponds to the *xy* comparison of the network of four treatments of Figure 1a. The set of vertices is *V* = { *x, y, u, v*} and the set of directed edges is *E* = { *xy, xv, vy, vu, uy*}. Flows *f _uv_* are given along the edges; their numerical values are equal to the respective absolute entries of ***h ^xy^*** and the direction of their corresponding edge is indicated in the subscript. As properties (a) to (d) imply, the arrows in Figure 1b indicate that the outflows of *x*, as well as the inflows of *y*, equal 1, and that the inflows equal the outflows in the intermediate nodes *u* and *v*.

### Streams

In Figure 1b there are three different paths from *x* to *y*, one based on direct evidence, { *xy*}, and two based on indirect evidence, { *xv, vy*} and { *xv, vu, uy*}. A path is a sequence of connected directed edges belonging to *E*, and we denote it as *π _i_*. As property (d) implies, each node occurs at most once in *π _i_*. Then, given the above properties of *f _uv_*, we can assign a flow *φ _i_* to each path *π _i_*. Flow *φ _i_* is equal to the smallest *f _uv_* in the path *π _i_*. Figure 1c shows the three paths from *x* to *y*; *π*_1_, *π*_2_ and *π*_3_, and their corresponding flows. Path *π*_1_ corresponds to *xy* and its flow, *φ*_1_, equals the flow of the single edge in path, *f _xy_* = 0.635. Path *π*_2_ is constituted from two edges, *xv* and *vy*; thus, flow *φ*_2_ = min( *f _xv_*, *f _vy_*) = 0.251. The flow corresponding to the third path *π*_3_ is *φ*_3_ = min( *f _xv_*, *f _vu_*, *f _uy_*) = 0.114.

We define a stream, *S _i_*, as the composition of a path and its associated flow, *S*_i_ = ( *φ _i_*, *π _i_*) with *i* = 1, ..., *I* where *I* is the total number of streams; here *I* = 3. Note that it holds $\sum_{i=1}^{I}\varphi_{i}=1$.

### Proportion contributions of direct comparisons

In order to assign proportion contributions to each direct comparison, we need to split each stream’s flow to the involved edges in the stream’s path. Equation 3 can be re-written as

$${\hat{\theta }}_{xy}^{N}=\varphi_{1}{\hat{\theta }}_{xy}^{D}+\varphi_{2}({\hat{\theta }}_{xv}^{D}-{\hat{\theta }}_{yv}^{D})+\varphi_{3}({\hat{\theta }}_{xv}^{D}-{\hat{\theta }}_{uv}^{D}-{\hat{\theta }}_{yu}^{D})\;\mathrm{Equation}5$$

with $\sum_{i=1}^{3}\varphi_{i}=1$. It follows from the properties of the elements of set ***F***, that each NMA effect can be written as a linear combination of direct and indirect effects, in the form of Equation 5. The effects are stochastically interdependent and, hence, their aggregation is different from the aggregation of studies in a pairwise meta-analysis.

To approximate the proportion contributions per comparison, we suggest dividing *φ _i_* by the length of the respective path *π _i_*, # *π _i_*. This will leave the proportion contribution of the direct evidence of the same treatment comparison equal to the diagonal of the ***H*** matrix and assign to each comparison involved in an indirect route a portion of the respective stream’s flow. Note that directed edges might be involved in more than one path; we thus define the proportion contribution of an edge *uv* as

$$p_{uv}=\sum_{\forall \;i\;\;\mathrm{where}\;uv\;\in \;\pi_{i}}\varphi_{i}/\#\pi_{i}\;\mathrm{Equation}6$$

Figure 1d shows the derivation of the proportion contributions of each direct comparison in the network of topical antibiotics. Hence, from the row of the ***H*** matrix (0.635, 0.365, –0.114, –0.251, –0.114), which shows the absolute contributions of the direct effects ${\hat{\theta }}_{xy}^{D},{\hat{\theta }}_{xv}^{D},{\hat{\theta }}_{yv}^{D},{\hat{\theta }}_{yu}^{D},{\hat{\theta }}_{uv}^{D}$ to ${\hat{\theta }}_{xy}^{N}$, we approximated their proportion contribution as 63.5%, 16.4%, 3.8%, 12.6% and 3.8%, respectively.

### Algorithm to decompose flows into proportion contributions

In this section, we present an iterative algorithm that generalizes the process outlined above to derive proportion contributions of each direct effect to the estimation of a ‘ *x* versus *y*’ NMA effect. We start by defining a graph *G _xy_* from ***h ^xy^***.

The algorithm is described as follows:

0. Set initial graph *G*_0_ = ( *V, E*_0_, *F*_0_) = *G _xy_*. *E*_0_ contains *uv* if *h _uv_* > 0 or contains *vu* if *h _uv_* < 0. The set of flows is *F*_0_ = { *f*_0, *uv*_ | *uv* ∈ *E*_0_}; numerical values of *f*_0, *uv*_ are equal to *h _uv_*.

Then, repeat the process below *I* times, equal to the number of streams in *G _i_*, until *E _i_* = {Ø}.

1. In *G*_*i*–1_, find the shortest path from *x* to *y*, *π _i_*, and define its flow as *φ _i_* = min{ *f*_*i*–1, *uv*_, *uv ∈ π _i_*}. Then, use *π _i_* and *φ _i_* to define the stream *S _i_* = ( *φ _i_*, *π _i_*).
2. Recalculate the flow of edges *uv* ∈ *π _i_* by subtracting *φ _i_* from the flow of the edges of the stream found: *f _i,uv_* = *f*_*i*–1, *uv*_ – *φ _i_* ∀ *uv* ∈ *π _i_*. The flow of the rest of the edges that do not belong to *π _i_* remain unchanged: *f _i,uv_* = *f*_*i*–1, *uv*_ ∀ *uv* ∉ *π _i_*.
3. Define *E _i_* as the set of edges *uv* for which *f _i,uv_* > 0; this is *E*_*i*–1_ after removing the edges with zero flow, *E _i_* = *E*_*i*–1_\{ *uv* | *f*_*i,uv*_ = 0}. Collect *f _i,uv_* to form the set *F _i_* = { *f _i,uv_* | *uv* ∈ *E _i_*}.
4. If *E _i_* ≠ {Ø} define *G _i_* = ( *V, E _i_, F _i_*) and go to step 1.

When the algorithm terminates, all streams *S _i_* = ( *φ _i_*, *π _i_*) have been identified and Equation 6 is used to derive the proportion contributions *p _uv_*.

Repeating the same process for all NMA effects, we derive all $p_{uv}^{xy}$ and collect them in a matrix ***P*** of the same dimensions as ***H***. The presented algorithm could be described as a reverse maximum flow Edmonds Karp algorithm ^8^, but instead of adding we remove augmenting paths.

It is possible that multiple shortest paths exist; in this case, the order in which one chooses such a path could in principle result in different proportion contributions per comparison. We can, thus, use the following modification in the algorithm to impose consistency. Instead of selecting the shortest path, we assign cost values *c _i,uv_* to each edge *uv* as follows: *c _i,uv_* = 2 – *f*_*i*–1, *uv*_. Then, we select the path from *x* to *y* with the minimum cost across comparisons included in *π _i_*. The definition of the cost values *c _i,uv_* assures that paths are selected from shortest to longest and removes any ambiguity regarding the selection of paths.

The starting point for the developed algorithm was a simplified version of the ***H*** matrix that does not consider the correlation induced by multi-arm trials. Alternatively, one could use the ***H*** matrix as described by König *et al.* ^4^ that extends the definition of the matrix for multi-arm designs. Note that any matrix whose rows can be interpreted as flow networks can be used as the starting point of the algorithm. The estimator of heterogeneity as well as the assumption of a different or common heterogeneity across comparisons in the network does not modify any aspect of the method.

Calculations in this paper were performed using R.

## Application

We apply the algorithm described above to the network of topical antibiotics ^7^.

### Proportion contributions of direct relative treatment effects to the estimation of the NMA effect between non-quinolone antibiotic and no treatment

Direct effects are obtained using the random effects model and the ***H*** matrix of dimension 6 *×*5 is calculated using Equation 2. The ***H*** matrix, along with NMA effects, is given in Table 1.

**Table 1.** *H* matrix in the network of topical antibiotics without steroids for chronically discharging ears. Columns correspond to direct comparisons and rows correspond to network meta-analysis (NMA) effects. Direct effects along with their variances and NMA effects with 95% confidence intervals (CIs) are given in the last column. Direct and NMA effects are measured as log odds ratios. **Positive values favour the first treatment.**

	*xy*	*xv*	*yu*	*yv*	*uv*	Direct effect	Variance of direct effect	NMA effect (95% CIs)
***xy***	0.635	0.365	–0.114	–0.251	–0.114	–2.29	0.42	–1.86 (–3.05,–0.67)
***xu***	0.603	0.397	0.632	–0.029	–0.368	–	–	–1.32 (–2.61,–0.02)
***xv***	0.545	0.455	0.170	0.375	0.170	0.35	0.63	–0.72 (–1.95,0.52)
***yu***	–0.032	0.032	0.745	0.223	–0.255	0.39	0.12	0.54 (–0.07,1.16)
***yv***	–0.090	0.090	0.284	0.627	0.284	1.24	0.15	1.14 (0.47,1.81)
***uv***	–0.058	0.058	–0.462	0.404	0.538	0.53	0.22	0.60 (–0.12,1.33)

***x***, no treatment; ***y***, quinolone antibiotic; ***u***, non-quinolone antibiotic; ***v***, antiseptic.

We begin by applying step 0 of the algorithm. We construct the network *G*_0_ = *G _xy_* = ( *V*, *E*_0_, *F*_0_) with source *x* and sink *y* corresponding to row ***h ^xy^***. The set of vertices is *V* = { *x, y, u, v*} and the set of directed edges, taking into account the signs of the elements of ***h ^xy^***, is *E*_0_ = { *xy, xv, vy, vu, uy*}. The set of flows is *F*_0_ = { *f*_0, *uv*_ | *uv* ∈ *E*_0_}, where *f*_0, *uv*_ equal the respective absolute values of Table 1 and are given along the edges of Figure 1b.

**Figure 2.**
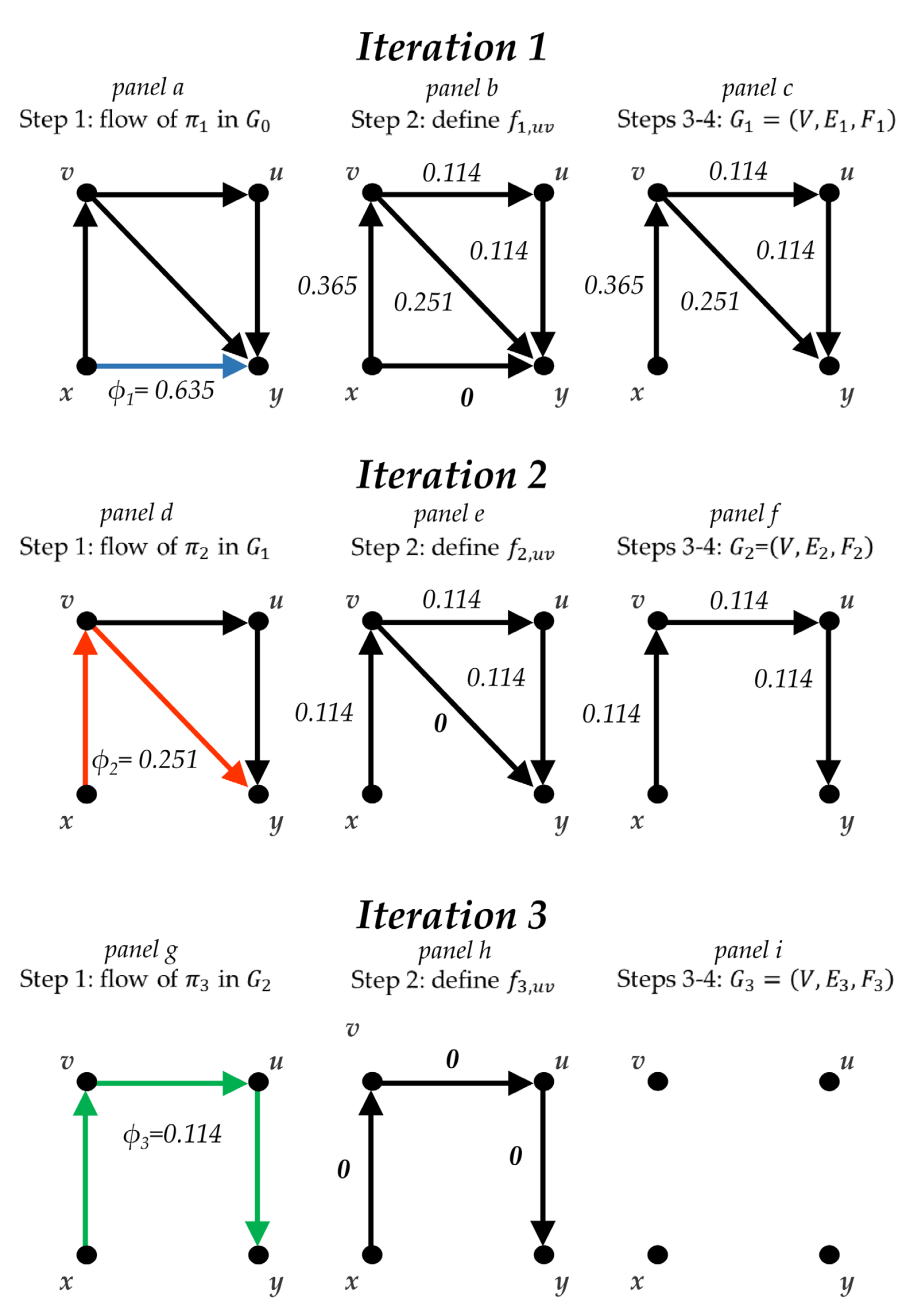
Illustration of the steps of the algorithm for approximating proportion contributions per comparison in the network of topical antibiotics without steroids for chronically discharging ears focusing on the comparison ‘ *x versus y*’. Treatment labels: *x*, no treatment; ***y***, quinolone antibiotic; ***u***, non-quinolone antibiotic; ***v***, antiseptic.

Then, we apply the developed iterative algorithm until *E _i_* = {Ø}. The iterations of the algorithm equal the number of existing streams from *x* to *y* and are illustrated in Figure 2.

### First iteration

1. In *G*_0_, find the shortest path from *x* to *y*, *π*_1_ = { *xy*}. Define its flow as *φ*_1_ = min{ *f*_0, *uv*_, *uv ∈ π*_1_} = *f*_0, *xy*_ = 0.635. Define stream *S*_1_ = ( *φ*_1_, *π*_1_) ( Figure 2a).
2. Recalculate the flow of edge *xy* ∈ *π*_1_ as *f*_1, *xy*_ = *f*_0, *xy*_ – *φ*_1_ = 0.635 – 0.635 = 0. The flow of the rest of the comparisons remains unchanged: *f*_1, *uv*_ = *f*_0, *uv*_ *∀ uv ∉ π*_1_ ( Figure 2b).
3. Define *E*_1_ as the set of edges *uv* for which *f*_1, *uv*_ > 0; edge *xy* is removed since its flow is zero, *E*_1_ = *E*_0_\{ *xy*}. Collect *f*_1, *uv*_ to form the set *F*_1_ = { *f*_1, *uv*_ | *uv* ∈ *E*_1_} ( Figure 2c).
4. Define *G*_1_ = ( *V, E*_1_, *F*_1_). As *E*_1_ = { *xv, vy, vu, uy*} ≠ {Ø}, go to step 1 ( Figure 2c).

### Second iteration

1. In *G*_1_, find the shortest path from *x* to *y*, *π*_2_ = { *xv,vy*}. Define its flow as *φ*_2_ = min{ *f*_1, *uv*_, *uv ∈ π*_2_} = *f*_1, *vy*_ = 0.251. Define stream *S*_2_ = ( *φ*_2_, *π*_2_) ( Figure 2d).
2. Recalculate the flow of edges *xv* and *vy* as *f*_2, *xv*_ = *f*_1, *xv*_ – *φ*_2_ = 0.365 – 0.251 = 0.114 and *f*_2, *vy*_ = *f*_1, *vy*_ – *φ*_2_ = 0.251 – 0.251 = 0. The flow of the rest of the comparisons remains unchanged: *f*_2, *uv*_ = *f*_1, *uv*_ *∀ uv ∉ π*_2_ ( Figure 2e).
3. Define *E*_2_ as the set of edges *uv* for which *f*_2, *uv*_ > 0; edge *vy* is removed since its flow is zero and thus *E*_2_ = *E*_1_\{ *vy*}. Collect *f*_2, *uv*_ to form the set *F*_2_ = { *f*_2, *uv*_ | *uv* ∈ *E*_2_} ( Figure 2f).
4. Define *G*_2_ = ( *V, E _2_, F*_2_). As *E*_2_ = { *xv, vu, uy*} ≠ {Ø}, go to step 1 ( Figure 2f).

### Third iteration

1. In *G*_2_, find the shortest path from *x* to *y*, *π*_3_ = { *xv, vu, uy*}. Define its flow as *φ*_3_ = min{ *f*_2, *uv*_, *uv ∈ π*_3_} = 0.114. Define stream *S*_3_ = ( *φ*_3_, *π*_3_) ( Figure 2g).
2. Recalculate the flow of edges *xv*, *vu* and *uy* as *f*_3, *xv*_ = *f*_3, *vu*_ = *f*_3, *uy*_ = *f*_2, *xv*_ – *φ*_3_ = 0.114 – 0.114 = 0 ( Figure 2h).
3. Define *E _3_* as the set of edges *uv* for which *f*_3, *uv*_ > 0; edges *xv*, *vu* and *uy* are removed since their flow is zero, *E*_3_ = *E*_2_\{ *xv, vu, uy*}. Collect *f*_3, *uv*_ to form the set *F*_3_ = { *f*_3, *uv*_ | *uv* ∈ *E*_3_} ( Figure 2i).
4. Define *G*_3_ = ( *V, E*_3_, *F*_3_). The set of direct edges is *E*_3_ = {Ø} and the algorithm is terminated at this point ( Figure 2i).

Figure 1c shows the flows of the three streams identified when applying the above algorithm. We then calculate the proportion contributions of each comparison to the ‘ *x* versus *y*’ NMA treatment effect estimate using Equation 6 ( Figure 1d). For instance, to calculate *p _xv_* we first have to identify the relevant paths; these were *π*_2_ and *π*_3_. Consequently,

$$p_{xv}=\frac{\varphi_{2}}{\left|\pi_{2}\right|}+\frac{\varphi_{3}}{\left|\pi_{3}\right|}=\frac{0.251}{2}+\frac{0.114}{3}=0.164=16.4\%$$

The calculations for deriving the proportion contributions of the other comparisons are shown in Table 2. Applying the algorithm to all NMA treatment effect estimates we get the entire proportion contribution matrix ***P***.

**Table 2.** Proportion contributions of direct comparisons to the ‘ *x* versus *y*’ network meta-analysis treatment effect in the network of topical antibiotics without steroids for chronically discharging ears.

	*xy*	*xv*	*yu*	*yv*	*uv*
***xy***	$p_{xy}=\frac{\varphi_{1}}{\left|\pi_{1}\right|}$ = 63.5%	$p_{xv}=\frac{\varphi_{2}}{\left|\pi_{2}\right|}+\frac{\varphi_{3}}{\left|\pi_{3}\right|}$ = 16.4%	$p_{yu}=\frac{\varphi_{3}}{\left|\pi_{3}\right|}$ = 3.8%	$p_{yv}=\frac{\varphi_{2}}{\left|\pi_{2}\right|}$ = 12.6%	$p_{uv}=\frac{\varphi_{3}}{\left|\pi_{3}\right|}$ = 3.8%

*x*, no treatment; *y*, quinolone antibiotic; *u*, non-quinolone antibiotic; *v*, antiseptic.

### Proportion study contributions

Matrix ***P*** ( Table 3) shows the proportion contributions of each direct comparison to each NMA treatment effect estimate. These proportions can be distributed to individual studies within each comparison according to their weights from direct meta-analyses. For example, *p*_*xy*_ = 63.5% and there are two studies examining the *xy* comparison. The individual study weights for the two studies are 0.69 and 1.54 resulting in study proportion contributions of $\frac{0.69}{0.69+1.54}63.5\%=19.6\%$ and $\frac{1.54}{0.69+1.54}63.5\%=43.8\%$ to the xy NMA treatment effect estimate. The application of this process to the entire matrix ***P*** leads to the matrix P* shown in Table 4. Adjusted weights as proposed by Rücker & Schwarzer ^9^ are used for multi-arm studies.

**Table 3.** Proportion contribution matrix *P* for the network of topical antibiotics without steroids for chronically discharging ears. Cells show the proportion contribution of direct comparisons indicated in the column to the network meta-analysis treatment effects indicated in the rows.

	*xy*	*xv*	*yu*	*yv*	*uv*
***xy***	63.5%	16.4%	3.8%	12.6%	3.8%
***xu***	30.1%	19.4%	31.1%	1%	18.4%
***xv***	24.4%	45.5%	5.7%	18.8%	5.7%
***yu***	1.1%	1.1%	74.5%	11.1%	12.2%
***yv***	4.5%	4.5%	14.2%	62.7%	14.2%
***uv***	1.9%	1.9%	22.1%	20.2%	53.8%

*x*, no treatment; *y*, quinolone antibiotic; *u*, non-quinolone antibiotic; *v*, antiseptic.

**Table 4.** Study proportion contribution matrix P* for the network of topical antibiotics without steroids for chronically discharging ears. Cells show the proportion contribution of individual studies indicated in the column to the network meta-analysis treatment effects indicated in the rows.

	Study 1	Study 2	Study 3	Study 4	Study 5	Study 6	Study 7	Study 8	Study 9	Study 10	Study 11	Study 12	Study 13
***xy***	19.7	63.4	2.8	0.5	0.6	0.4	0.6	0.7	2.7	1.3	0.8	2.3	4.2
***xu***	9.3	40.4	8.8	4.4	5.3	3.5	5.1	5.4	6.9	6.5	3.9	0.2	0.3
***xv***	7.6	67.1	4.2	0.8	1	0.6	0.9	1	4.1	2	1.2	3.4	6.2
***yu***	0.3	4.63	13.6	10.5	12.7	8.4	12.2	12.9	12.2	4.3	2.6	2	3.7
***yv***	1.4	23.5	12.1	2	2.4	1.6	2.3	2.4	12.2	5	3	11.3	20.7
***uv***	0.6	8.4	19	3.1	3.8	2.5	3.6	3.8	14.4	19.1	11.5	3.6	6.7

*x*, no treatment; *y*, quinolone antibiotic; *u*, non-quinolone antibiotic; *v*, antiseptic.

### Using proportion study contributions to quantify the impact of a characteristic in a direct comparison

**Figure 3.**
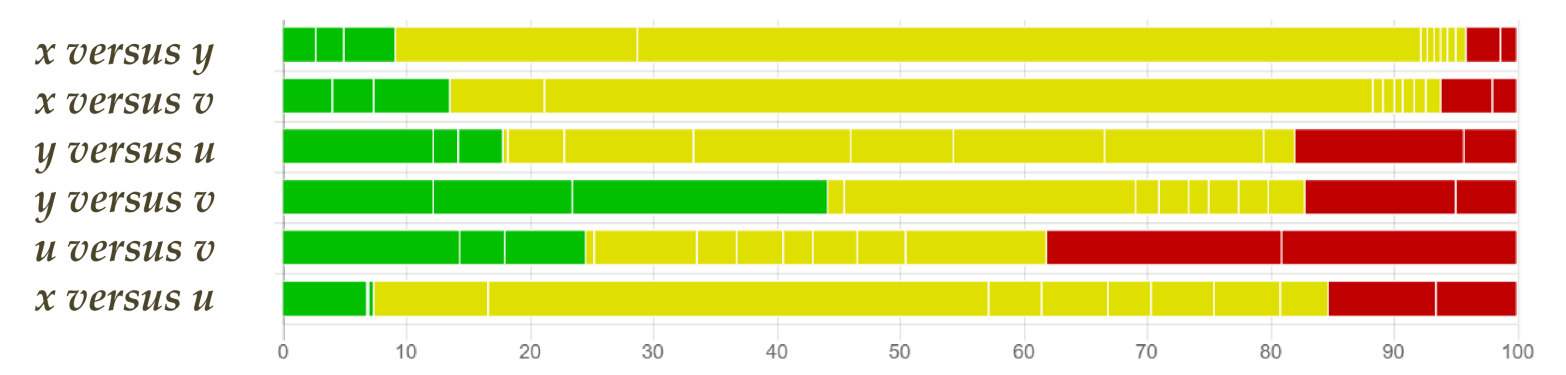
Bar plot showing the study proportion contributions of direct comparisons with low (green), moderate (yellow) and high (red) risk of bias. The bar plot has been produced in CINeMA (Confidence In Network Meta-Analysis) software ^11^. Studies are synthesized using the random effects model. ***x***, no treatment; ***y***, quinolone antibiotic; ***u***, non-quinolone antibiotic; ***v***, antiseptic.

The algorithm translating the ***H*** matrix into study proportion contributions can be applied to quantify the influence that a study-level characteristic has in the estimation of the NMA effects. For instance, if risk of bias judgements for individual studies are available, we can obtain an approximation of the proportion of each NMA treatment effect estimate that is coming from studies with a ‘high’, ‘moderate’, or ‘low’ risk of bias. Salanti *et al*. suggested the visualisation of this information using a bar plot, in which direct comparisons of the same risk of bias level have been grouped ^3^. Figure 3 shows such a bar plot using the algorithm described in this paper and distributing comparison proportion contributions to study proportion contributions; inspecting Figure 3 can support judgements regarding the importance of study limitations for different NMA treatment effect estimates. For instance, studies with high risk of bias contribute more than 50% in the estimation of the ‘ *u* versus *v*’ comparison, potentially reducing the confidence that we can place in this particular NMA treatment effect estimate.

### Proportion contributions of direct comparisons in a large complex network of interventions

**Figure 4.**
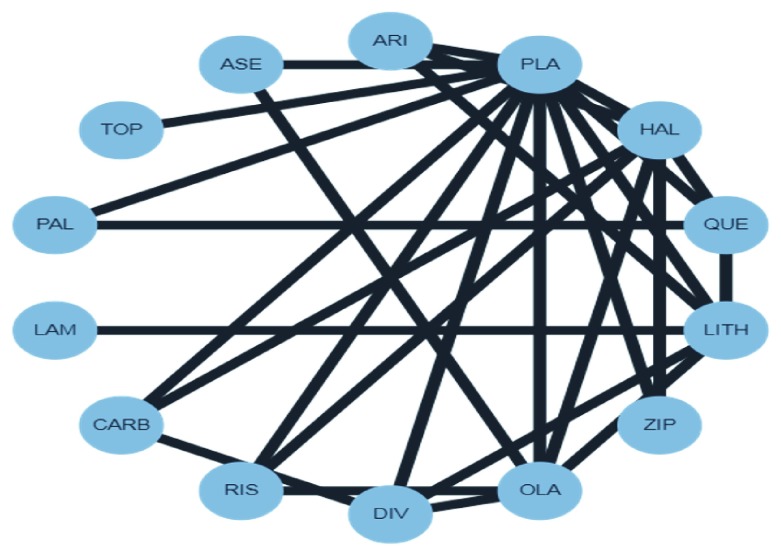
Network plot for the network of antimanic drugs. ASE, asenapine; ARI, aripiprazole; PLA, placebo; HAL, haloperidol; QUE, quetiapine; LITH, lithium; ZIP, ziprasidone; OLA, olanzapine; DIV, divalproex; RIS, risperidone; CARB, carbamazepine; LAM, lamotrigine; PAL, paliperidone; TOP, topiramate; ASE, asenapine.

So far, we have illustrated how to derive proportion contributions for a network with four treatments. However, the algorithm can be straightforwardly applied to large networks of any structure, as soon as the involved treatments are connected. Consider for example a large network examining antimanic drugs ( Figure 4) ^10^. Let us concentrate on the comparison PLA versus OLA (‘placebo versus olanzapine’); the algorithm starts by applying step 0 and constructing network *G*_0_. Then, we continue by finding the shortest path in the first iteration, which corresponds to the direct comparison, and define its flow and stream *S*_1_ = ( *φ*_1_, *π*_1_). The number of algorithm’s iterations is equal to the number of streams from placebo to olanzapine, which turns out to be 16. The resulting entire proportion matrix is given in Supplementary File 2.

Outcome data from the example network of topical antibiotics for the treatment of chronic otitis media with ear discharge in patients with eardrum perforations ^7^ Data labels: study, name of individual studies; id, id of the individual studies; t; treatment; r, number of events; n, sample size; rob, risk of bias per study. Click here for additional data file. Copyright: © 2018 Papakonstantinou T et al. 2018 Data associated with the article are available under the terms of the Creative Commons Zero "No rights reserved" data waiver (CC0 1.0 Public domain dedication).

## Discussion

In this paper, we present a new approach to derive proportion contributions of individual studies to the treatment effect estimates in NMA. We made use of the fact that the composition of network treatment effect estimates can be interpreted as a flow of evidence. An assumption that underlies our algorithm is the equal split of the stream flow to the involved comparisons. Although indirect effects are not weighted averages, we find this approximation to be a pragmatic approach that reasonably reflects the amount that each comparison contributes to network effects. The derivation of the contribution of sources of evidence in a Bayesian NMA is not straightforward, as no analogue to the **H** matrix exists. The application of the method described in this paper, however, can be used to derive useful approximations of the contributions of studies.

Applying the algorithm to networks of interventions can be used to quantify the contribution of potential study limitations to the NMA treatment effect estimates. Study limitations may lead to biased NMA treatment effect; however, the amount and direction of bias in the NMA treatment effect as a result of the within-study bias is not straightforward to define and is not currently accommodated within the proportion contribution matrix. First, a single biased trial may affect an entire indirect route; thus, even if its proportion contribution is small, its consequences in the estimation of the NMA treatment effect may be important. Second, the direction of bias across studies involved in a stream may vary. For example, bias in two comparisons in the same stream may either cancel out or add-up in favor of one of the two treatments. We aim to extend the methods presented in this paper to develop a network meta-regression model that will use the direction and the amount of bias to determine whether and how much NMA treatment effect estimates will be biased as the result of within-study bias.

Alternative methods to derive the relative contribution of all sources of evidence have been developed ^12, 13^. Krahn *et al.* define influence functions to describe the extent to which changes in study effects would be translated into NMA treatment effects ^13^. An alternative approach, based on the decomposition of Fisher’s information matrix, has been proposed to derive proportion study weights in a variety of meta-analysis models, including meta-regression, network meta-analysis and individual patient data meta-analysis ^12^. Further investigation of the degree of agreement between our algorithm and that of Riley *et al.* ^12^ would be of interest.

In the example implemented in the Application, there is no other possible set of paths, and associated streams, that could be selected from *x* to *y* in order to partition the inflow of *x*: *π*_1_, *π*_2_ and *π*_3_ is the only possible set of streams ( Figure 1c). Thus, even if we were taking paths using different criteria, i.e. from longest to shortest, according to values from the ***H*** matrix or even randomly, the proportion contributions given in Table 2 would be identical. However, cases exist where the selection of paths does influence the derivation of the ***P*** matrix. In Supplementary File 3, we elaborate on the selection of direct paths in the algorithm and discuss some alternative modifications of the algorithm. We are planning to examine the properties of the different approaches in greater detail in a follow up project.

We offer an R package ^14^, which we also use in the software application CINeMA (Confidence In Network Meta-Analysis) ^11^, that aims to simplify the evaluation of confidence in the findings from NMA. While CINeMA largely follows the framework previously developed by Salanti *et al*. ^3^, the refinement of several methodological aspects is currently under development. Core aspects of the approach include the consideration of the relative contributions of each direct comparison to each NMA treatment effect estimate. To this end, CINeMA uses the proportion contribution matrix as described in this paper. The command *netweight* in Stata has also been updated to use the described approach.

We believe that the approach described in this paper is a useful and novel addition to network meta-analysis methodology, which allows the consistent derivation of the proportion contributions of direct evidence from individual studies to network treatment effects.

## Data availability

The data referenced by this article are under copyright with the following copyright statement: Copyright: © 2018 Papakonstantinou T et al.

Data associated with the article are available under the terms of the Creative Commons Zero "No rights reserved" data waiver (CC0 1.0 Public domain dedication). 

**Dataset 1: Outcome data from the example network of topical antibiotics for the treatment of chronic otitis media with ear discharge in patients with eardrum perforations ^7^.** Data labels: study, name of individual studies; id, id of the individual studies; t; treatment; r, number of events; n, sample size; rob, risk of bias per study. DOI: 10.5256/f1000research.14770.d203174 ^15^.

## Supplementary material

**Supplementary File 1. Proof that the sum of all outflows from the source is equal to the sum of all inflows to the sink and both are 1.**

**Supplementary File 2. Proportion contribution matrix *P* for the network of antimanic drugs. Cells show the proportion contribution of direct comparisons indicated in the column to the network meta-analysis treatment effects indicated in the rows.** ASE, asenapine; ARI, aripiprazole; PLA, placebo; HAL, haloperidol; QUE, quetiapine; LITH, lithium; ZIP, ziprasidone; OLA, olanzapine; DIV, divalproex; RIS, risperidone; CARB, carbamazepine; LAM, lamotrigine; PAL, paliperidone; TOP, topiramate; ASE, asenapine.

**Supplementary File 3. Considerations on the selection of streams**
