# Supplementary material for: Estimating the contribution of studies in network meta-analysis: paths, flows and streams
Source: F1000Res. 2018 Dec 13;7:610. Originally published 2018 May 18. [Version 3] doi: 10.12688/f1000research.14770.3 (PMC6148216; doi:10.12688/f1000research.14770.3)
Supplement: Supplementary file 2 [file f1000research-7-19069-s0001.tgz › 94cb42a7-1f60-44a3-ba5d-005cb03ce311.pdf]

Flows and H matrix elements

Consider a network meta-analysis with hat matrix $\mathbf{H}$ given by

$$\mathbf{H} = \mathbf{X}\ (\mathbf{X}^\top \mathbf{W} \mathbf{X})^+ \mathbf{X}^\top \mathbf{W}$$

where $\mathbf{X}$ is the (full) design matrix in the sense of (Rücker and Schwarzer 2014), $\mathbf{W}$ is the weight matrix (already adjusted for multi-arm studies, if necessary), and the treatments (nodes) and comparisons (edges) are lexically ordered such that we without loss of generality may consider the first treatment and the first comparison involving this treatment. We select the first row of $\mathbf{H}$ (corresponding to the first comparison) by

$$\mathbf{e}_1^\top \mathbf{H}$$

(where e_1 is the first unit vector in the row space of $\mathbf{H}$) and are interested in the sum of those elements that correspond to outflows from node 1 (the first treatment). These correspond to the first column of $\mathbf{X}$, that is

$$\mathbf{X}\mathbf{u}_1$$

where $\mathbf{u}_1$ is the first unit vector in the column space of $\mathbf{X}$. Together, because of $\mathbf{X}\ (\mathbf{X}^\top \mathbf{W} \mathbf{X})^+ (\mathbf{X}^\top \mathbf{W} \mathbf{X}) = \mathbf{X}$ (Rücker and Schwarzer 2014, Appendix A2) we have

$$\mathbf{e}_1^\top \mathbf{H}\ \mathbf{X}\mathbf{u}_1 = \mathbf{e}_1^\top \mathbf{X}\ (\mathbf{X}^\top \mathbf{W} \mathbf{X})^+ (\mathbf{X}^\top \mathbf{W} \mathbf{X})\ \mathbf{u}_1 = \mathbf{e}_1^\top \mathbf{X}\ \mathbf{u}_1 = 1$$

which is the top left element of $\mathbf{X}$. Thus the sum of outflows from node 1 is 1. This result was given without proof in (König, Krahn, and Binder 2013).

Remark 1 Due to our construction, the elements of $\mathbf{e}_1^\top \mathbf{H}$ that start from node 1 are all positive. When showing that they add to one we have shown that they lie in $[0, 1]$.

Remark 2 Using a similar argument we can show that all inflows into the last node are positive and sum up to one.

Remark 3 As the nodes can be arbitrarily ordered, the same arguments hold to all other nodes (outflows and inflows). Note that when fixing a certain order, the signs of other matrix elements may change, but their absolute values are restricted to the interval $[-1, 1]$.

References

König, J., U. Krahn, and H. Binder (2013). Visualizing the flow of evidence in network meta-analysis and characterizing mixed treatment comparisons. *Statistics in Medicine 32*(30), 5414–5429. doi: 10.1002/sim.6001.

Rücker, G. and G. Schwarzer (2014). Reduce dimension or reduce weights? Comparing two approaches to multi-arm studies in network meta-analysis. *Statistics in Medicine 33*, 4353–4369. DOI: 10.1002/sim.6236.
