# Supplementary material for: Estimating the contribution of studies in network meta-analysis: paths, flows and streams
Source: F1000Res. 2018 Dec 13;7:610. Originally published 2018 May 18. [Version 3] doi: 10.12688/f1000research.14770.3 (PMC6148216; doi:10.12688/f1000research.14770.3)
Supplement: Supplementary file 4 [file f1000research-7-19069-s0003.tgz › 58127715-9033-4ef8-8b90-5908afe3091e_Supplementary_file_3.docx]

# Considerations on the selection of streams

## Number of directed paths

Consider the graph $G_{xy}=(V,E,F)$ as defined in section ‘Comparison graph’ and denote $\Pi$ the set of *all* directed paths existing from source $x$ to sink $y$. In the network of topical antibiotics $\Pi$ has three elements, $\left\{ xy \right\}$, $\left\{ xv,vy \right\}$ and $\left\{ xv,vu,uy \right\}$. However, in the general case, the number of paths used in the algorithm is smaller than the number of elements of $\Pi$. The number of directed paths which suffice to ‘spend’ the flow from source to sink is less or equal to $df + 1$ where $df$ is the inconsistency degrees of freedom in the Lu and Ades inconsistency model (1), $df=D-T+1$.

The number of directed paths which suffice to ‘spend’ the flow from source to sink can be less than $df + 1$ for some comparisons if a network involves one or more edges whose deletion would lead to two disconnected subnetworks; we will denote such edges as *bridges*. If no bridges exist in the network, the number of directed paths is equal to $df + 1$. Consider, for example, the two hypothetical networks shown in Appendix Figure 1. No bridge exists in the network shown in panel a of Appendix Figure 1 and the number of directed paths for any comparison in the network is 4 which is equal to $df + 1=7-5+1+1$. In the example shown in panel b of Appendix Figure 1, however, comparison $cd$ constitutes a bridge. While there are three paths, equal to $df + 1=3$, for some comparisons corresponding to NMA effect estimates (e.g. for ‘$a$ versus $f$’) we have only two directed paths from $a$ to $b$, $\{ab\}$ and $\{ac,cb\}$ , i.e. the ‘$a$versus $b$’ NMA effect estimate is informed only by the $abc$ part of the network. Thus, in networks with one or more bridges the number of paths between pairs of treatments within one part of the network depends on the degrees of freedom within this part only, ignoring the remaining network. A special case of a bridge in a network occurs in comparisons which are informed only by direct evidence.


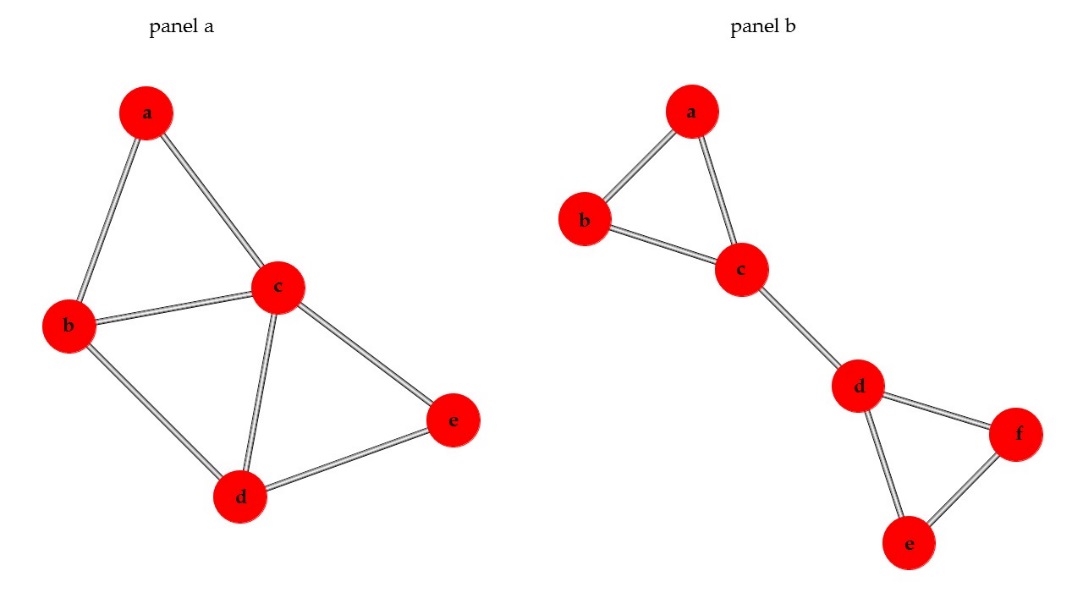


Appendix Figure 1. Two hypothetical networks illustrating the number of directed paths in networks without (panel a) or with (panel b) a bridge.

Another situation where the number of directed paths which suffice to ‘spend’ the flow from source to sink is less than $df + 1$ elements occurs when a vertex has at least two inflows and two outflows, all equal between them. In such a situation the flow from source to sink may be spent in less than $df + 1$ directed paths. Such a vertex will be called *breaking vertex*. Note that an NMA treatment effect estimate for treatments separated by a bridge or a breaking vertex (say treatments $x$ and $y$) can be seen as an “indirect” comparison through either the intermediate bridge (constituting by treatments $b_{1}$ and $b_{2}$) or the intermediate breaking vertex $\left( b_{0} \right)$.

In particular, in the case of a bridge, the NMA treatment effect estimate is derived as

$$\hat{\theta}_{xy}^{N}=\hat{\theta}_{xb_{1}}^{N}+\hat{\theta}_{b_{1}b_{2}}^{N}+\hat{\theta}_{b_{2}y}^{N}$$

and in the case of a breaking vertex the NMA treatment effect estimate is

$$\hat{\theta}_{xy}^{N}=\hat{\theta}_{xb_{0}}^{N}+\hat{\theta}_{b_{0}y}^{N}$$

It is, thus, implied that in such a case the separate subnetworks contribute equally to the estimation of $\hat{\theta}_{xy}^{N}$.

## Ambiguity due to non-unique set of directed paths

Consider again the network shown in panel a of Appendix Figure 1 and let us focus on the comparison ‘$a$ versus $e$’ for which there is no direct evidence. The set $\Pi$ contains 5 paths; $\left\{ ac, ce \right\}$, $\left\{ ab,bc,ce \right\}$, $\left\{ ab,bd,de \right\}$, $\left\{ ac,cd,de \right\}$, $\left\{ ab,bc,cd,de \right\}$ while the number of paths which suffice to ‘spend’ the flow from source to sink is 4. However, the set of directed paths to be selected is not unique. One can start the partition of the flow selecting the shortest path, as we have done so far, or taking another selection process. Consider for example Appendix Figure 2 which shows the comparison graph $G_{ae}$ along with the selection of paths and the associated derivation of percentage contributions in two different ways. This example shows two different ways of partitioning stream flows to paths illustrating that there is not a unique set of directed paths of $G_{ae}$. The two different approaches result to different percentage contributions, with the largest difference in percentage contributions occurring for the ‘$c$ versus $e$’ comparison (23% versus 25%).


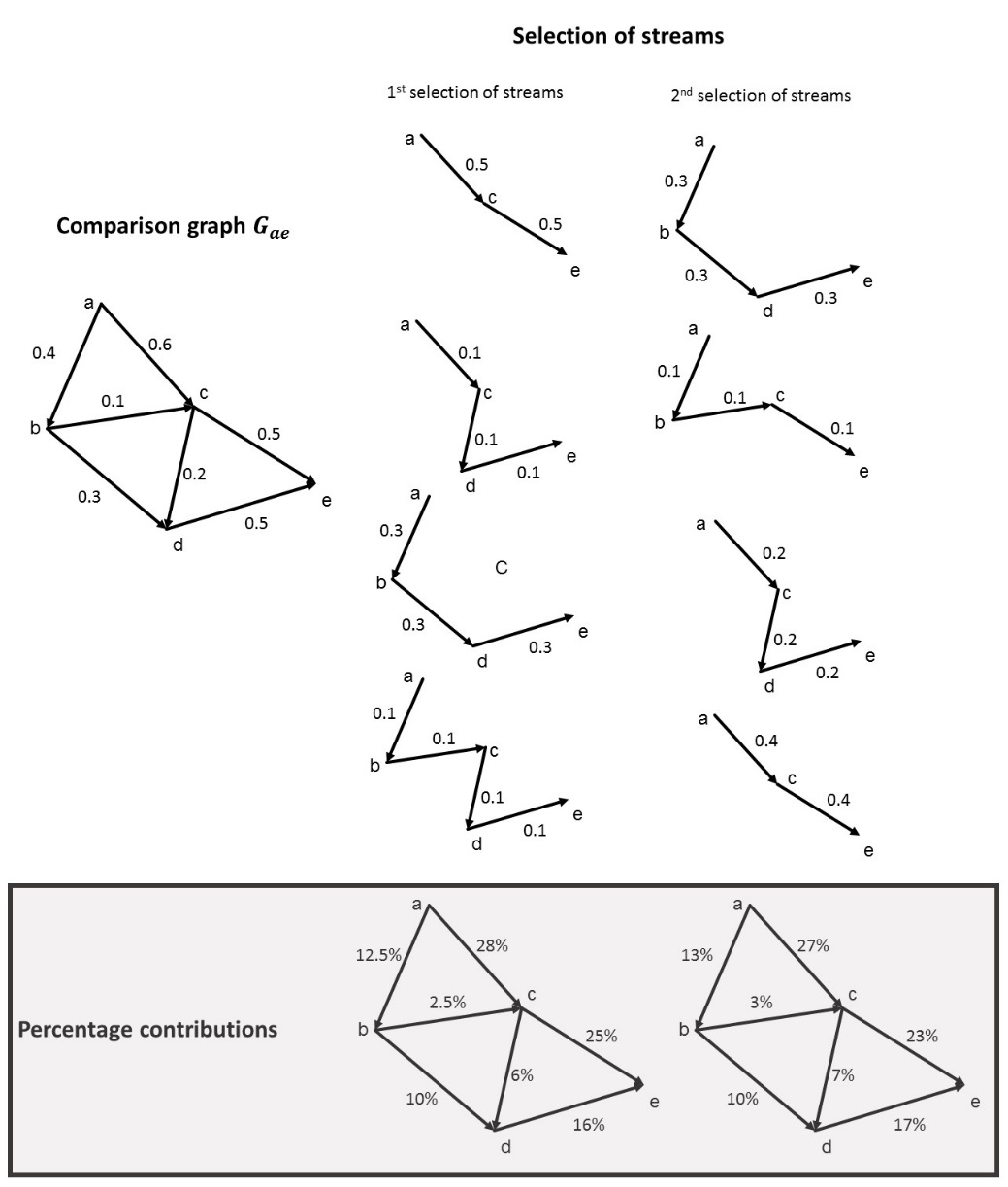


Appendix Figure 2. Hypothetical network illustrating the non-unique way of defining directed paths.

## Alternative strategies

As it was shown in the example of Appendix Figure 2, there is not a unique set of paths from source to sink that can be sequentially taken until the source’s inflow is 0. This finding reveals an ambiguity in the selection of paths to be taken. Apart from the ‘shortest path’ approach, several alternative strategies in selecting the directed paths in the algorithm could be considered. We present below some possible modifications of the algorithm presented in this paper.

1. Select paths randomly: This algorithm differs from the shortest path algorithm in step 1. Instead of finding the shortest path from $x$ to $y$, the algorithm finds randomly a neighbour node, say $u$, where each neighbour node has equal probability of being selected. Then, from $u$, the algorithm finds randomly the next neighbour node and the process continues until the path gets to sink. Then, this path is $\pi_{i}$ whose flow is$\varphi_{i}=\min\left\{ f_{i-1,uv}, uv \in\pi_{i} \right\}$ and stream $S_{i}=\left( \varphi_{i},\pi_{i} \right)$ is defined. The process is repeated until all flow is spent as in the shortest path algorithm.
2. Take the average of many randomly selected paths: This algorithm constitutes of implementing ‘algorithm a’ multiple times and taking the average percentage contributions across comparisons.
3. Select paths according to their flow: This algorithm starts with the path with the smallest flow and then stepwise chooses paths with the smallest flow of the remaining network. This approach is similar to the approach of assigning costs in the shortest path algorithm when equally lengthy paths exist. It is limited by the fact that the order depends on the values of the $\boldsymbol{H}$ matrix and not only the network structure. Moreover, it requires that all directed paths are found before the selection of streams.

## Comparison of alternative strategies

It would be of interest to examine the impact of the selection of paths and to choose among the described alternative strategies and we plan such work in the future. In general, the shortest path algorithm gives precedence to the shortest paths and thus it might be preferable in some contexts where longer chains of evidence are considered less trustworthy. For instance, in Appendix Figure 2 where the 1^st^ selection of streams represents the shortest path algorithm and the 2^nd^ selection of streams could represent algorithm a as it is a random selection, the shortest path $\{ac,ce\}$ path gets a flow of 0.4 with algorithm a, compared to a flow of 0.5 attributed to the particular path taking the shortest path first. Of interest, algorithm c gives the same results with the shortest path algorithm while the paths are found in a different order $\left\{ ab,bc,cd,de \right\}$, $\left\{ ac,cd,de \right\}$,$\left\{ ab,bd,de \right\}$, $\left\{ ac, ce \right\}$.

# References

1. Lu G, Ades AE. Assessing Evidence Inconsistency in Mixed Treatment Comparisons. J Am Stat Assoc. 2006 Jun 1;101(474):447–59.
