# Supplementary material for: Estimating the contribution of studies in network meta-analysis: paths, flows and streams
Source: F1000Res. 2018 Dec 13;7:610. Originally published 2018 May 18. [Version 3] doi: 10.12688/f1000research.14770.3 (PMC6148216; doi:10.12688/f1000research.14770.3)
Supplement: Peer review history

# Peer review: Reviewer response for version 1

**DOI:** 10.5256/f1000research.16071.r37273

**Published:** 2018-08-16

**Authors:** John R. Thompson

**Affiliations:** 1 Department of Health Sciences, University of Leicester, Leicester, UK

This report is also available as a separate PDF.

 I have no special knowledge of the measurement of information flow in a network meta-analysis and so I read this paper as a biostatistician with a general interest in meta-analysis.

 I like the idea of associating a descriptive measure of importance to each edge in a network and the authors of this paper present an interesting step in that direction, though my feeling is that this proposal will not turn out to be the final solution. I have two areas of concern, one is to do with terminology and the other with the scope of applicability of the proposal solution.

 In criticising the terminology, I have to accept that the authors are largely following common practice and so my criticisms are partly aimed at the meta-analysis community. None the less there were three things that irritated me. Firstly, throughout the paper, proportions are referred to as percentages. Next, the title of the paper says it is about "the contribution of studies in network meta-analysis" while actually it is about the contribution of different effect estimates. Finally, the measure adopted is the weight given to each effect estimate when calculating the pooled estimate. In this paper and elsewhere in the meta-analysis literature, this weight is called a contribution. Perhaps I am being too pedantic but the weight and contribution are different ideas and it does not help with the terminology confuses them. 

 Now the proposed method. Taking the example from the paper, the authors note that the pooled estimate of the xy effect can be calculated using equation 1, where *θ̂ ^I^_xvy _*is the indirect estimate of xy along the path xy. Further it is true, at least for this example, that Σ *L*_*iφi* _= 1 where L _i_ is the length of the path. So we can attach the weights *φ* to the edges of the network, sum them when an edge contributes to more than one estimate and the resulting weights will sum to one over the whole network. 

 This argument works for the example presented in the paper but it is not clear to me what conditions have to hold for it to work generally. The authors note in the paper that including a multi-arm trial in the meta-analysis would cause a problem, presumably because some of the direct or indirect estimates would not be independent. Are there any other conditions that have to hold? For example, can we have any structure of random effects in the meta-analysis model? What about the Bayesian models that are often used for network meta-analysis?

 The authors take the matrix, H, which projects individual estimates such as xy, xy, uy, etc. into their predicted values under the meta-analysis model and they present an algorithm for converting those values into weights that are equivalent to the *φ*'s.The algorithm is sensible and works for the simple example in the paper but one is again left wondering whether or not it works under all circumstances. After all, the algorithm is presented without any proof that it works.

 My own feeling is that a contribution is best measured by the sledgehammer approach of analysing the network with and without a particular edge, but the authors' suggested approach is much less computationally demanding and I think that it would be appreciated by many applied researchers provided they were certain that it could be safely used with their particular network and their particular meta-analysis model.

I confirm that I have read this submission and believe that I have an appropriate level of expertise to confirm that it is of an acceptable scientific standard.

# Response

**Published:** 2018-12-05

**Authors:** Adriani Nikolakopoulou

**Affiliations:** Institute of Social and Preventive Medicine, Switzerland

We are grateful to Dr. John R. Thompson for his time and valuable comments and for recommending approval of our paper. We believe that we have been able to address all the comments raised and we have prepared a next version (Version 3) of the paper. Below are the detailed replies to reviewer’s comments; our responses to the first reviewer, Dr. Jochem König, can be found in https://f1000research.com/articles/7-610/v2.

**Referee Report 16 Aug 2018**

**John R. Thompson**, Department of Health Sciences, University of Leicester, Leicester, UK 

**Approved**

**I have no special knowledge of the measurement of information flow in a network meta-analysis and so I read this paper as a biostatistician with a general interest in meta-analysis.**

**I like the idea of associating a descriptive measure of importance to each edge in a network and the authors of this paper present an interesting step in that direction, though my feeling is that this proposal will not turn out to be the final solution. I have two areas of concern, one is to do with terminology and the other with the scope of applicability of the proposal solution.**

**In criticising the terminology, I have to accept that the authors are largely following common practice and so my criticisms are partly aimed at the meta-analysis community. None the less there were three things that irritated me. Firstly, throughout the paper, proportions are referred to as percentages. Next, the title of the paper says it is about "the contribution of studies in network meta-analysis" while actually it is about the contribution of different effect estimates. Finally, the measure adopted is the weight given to each effect estimate when calculating the pooled estimate. In this paper and elsewhere in the meta-analysis literature, this weight is called a contribution. Perhaps I am being too pedantic but the weight and contribution are different ideas and it does not help with the terminology confuses them.**

Thank you for these interesting notes on meta-analysis terminology. At first, we agree that referring to “percentage contributions” might not be fitting and calling them “proportions” is more appropriate. We, thus, replaced throughout percentages with proportions.

Regarding the reporting of contribution of studies in the title of our paper, we have extended the method to quantify the contribution of studies as suggested by the first reviewer, Dr. Jochem König (comment 6). Thus, we left the title of the paper unmodified.

Weights and contributions are indeed sometimes used interchangeably. In this paper, however, we believe that the definition of matrix and the set of flows render the distinction clear. We only talk about “weights” of paths in the Introduction, where we have not yet explained the terminology to be used and thus it is convenient describing the elements of matrix as generalized weights from pairwise meta-analysis. We apologise in advance if we have misunderstood your comment.

**Now the proposed method. Taking the example from the paper, the authors note that the pooled estimate of the xy effect can be calculated using equation 1, where *θ̂ ^I^_xvy _*is the indirect estimate of xy along the path xy. Further it is true, at least for this example, that Σ *L _iφi_*** **= 1 where L _i_ is the length of the path. So we can attach the weights *φ* to the edges of the network, sum them when an edge contributes to more than one estimate and the resulting weights will sum to one over the whole network.**

**This argument works for the example presented in the paper but it is not clear to me what conditions have to hold for it to work generally. The authors note in the paper that including a multi-arm trial in the meta-analysis would cause a problem, presumably because some of the direct or indirect estimates would not be independent. Are there any other conditions that have to hold? For example, can we have any structure of random effects in the meta-analysis model? What about the Bayesian models that are often used for network meta-analysis?**

Thank you for this insightful comment. We have clarified at the beginning of the Methods section that we consider *“a simplified version of the  matrix described by König et al. *[1] *that does not take into account the correlation induced by multi-arm trials.”*

In this case, and considering the properties of the elements of set  it always holds that Equation 3 can be re-written as Equation 5. We now clarify in the subsection ‘Proportion contributions of direct comparisons’:

*“It follows from the properties of the elements of set  that each NMA effect can be written as a linear combination of direct and indirect effects, in the form of Equation 5”.*

We clarify at the end of ‘Algorithm to decompose flows into percentage contributions’ section that multi-arm studies can be handled if a modified  matrix is used. We also clarify that the structure of random effects does not play a role in the calculations. In particular, we write:

*“The starting point for the developed algorithm was a simplified version of the matrix* *that does not consider the correlation induced by multi-arm trials. Alternatively, one could use the matrix as described by König et al. *[1] *that extends the definition of the matrix for multi-arm designs. Note that any matrix whose rows can be interpreted as flow networks can be used as the starting point of the algorithm. The estimator of heterogeneity as well as the assumption of a different or common heterogeneity across comparisons in the network does not modify any aspect of the method.”*

Within a Bayesian framework, the matrix is not defined. We added in the Discussion:

*“The derivation of the contribution of sources of evidence in a Bayesian NMA is not straightforward, as no analogue to the matrix exists. The application of the method described in this paper, however, can be used to derive useful approximations of the contributions of studies.”*

**The authors take the matrix, H, which projects individual estimates such as xy, xy, uy, etc. into their predicted values under the meta-analysis model and they present an algorithm for converting those values into weights that are equivalent to the *φ*'s.The algorithm is sensible and works for the simple example in the paper but one is again left wondering whether or not it works under all circumstances. After all, the algorithm is presented without any proof that it works.**

Thank you for pointing this out. The algorithm we present in the ‘Algorithm to decompose flows into proportion contributions’ is described for the general case of a network of interventions containing any number of studies, treatments and loops. Indeed, we do not present a proof that it works under all circumstances. We have, however, applied the algorithm in numerous NMA applications apart from the simple example used in the results of this paper. Moreover, as mentioned in the Discussion, the web application CINeMA (Confidence In Network Meta-Analysis) [2], that aims to simplify the evaluation of confidence in the findings from NMA, uses the proportion contribution matrix as described in this paper. Users of CINeMA have not reported any case that the algorithm does not work. We are planning to examine the properties of the different approaches on the selection of direct paths in the algorithm in a follow up project (preliminary work is described in Supplementary File 3), which could be combined with a proof that the algorithm (and its modifications) works.

**My own feeling is that a contribution is best measured by the sledgehammer approach of analysing the network with and without a particular edge, but the authors' suggested approach is much less computationally demanding and I think that it would be appreciated by many applied researchers provided they were certain that it could be safely used with their particular network and their particular meta-analysis model.**

We totally agree that such ‘leave one out’ approaches would be also of interest. Relevant methods have been proposed by Krahn et al. [3] for contribution to the effect and by Riley et al. [4] for contribution to the variance. The former is useful in measuring how much a direct effect could bias the NMA effect and thus is not relevant in this case where we opted for a representation of contribution of pieces of evidence according to a study characteristic. The latter is based on the decomposition of Fisher’s information matrix and is associated with some issues that need further investigation.

We had already mentioned and discussed the Riley et al. approach in the Discussion. We now added:

*“Krahn et al. define influence functions to describe the extent to which changes in study effects would be translated into NMA treatment effects *[3] *.”*

# Peer review: Reviewer response for version 1

**DOI:** 10.5256/f1000research.16071.r36470

**Published:** 2018-08-14

**Authors:** Annette M. O'Connor

**Affiliations:** 1 Department of Veterinary Diagnostic and Production Animal Medicine, College of Veterinary Medicine, Iowa State University, Ames, IA, USA

This paper described an updated approach to deriving the percentage contributions of the direct comparison used in treatment estimates. In the interests of full disclosure, I am not a statistician and was reviewing from the view point of an applied user of NMA. There are several papers on this topic in recent years, and it is an active body of work in this area. The article is straightforward to read, although made considerably more readable if one is aware of the content in references, 3,4 and 5. 

 I have to admit that this is an example of why I appreciate the open review system. I enjoyed the paper and found it easy to read and follow, but the others reviewer's comments were a significant contribution also. I don't have any additional critique of the proposed approach, but I look forward to seeing how the investigators address those or provided some discussion, as this would help the less statistically inclined reader like me. Again, the reason I wanted this, is that I am an applied user and such discussions are very helpful. In particular, I would like to have seen some discussion of the 1st comment. I would also like to see if the comment about Figure 3 could be incorporated - perhaps not feasible. 

 Concerning the approach to calculating the % contribution and how to weight the flows, I came away with the impression that the decision of equal split of the stream flow as arbitrary (perhaps not precisely the correct term) or perhaps pragmatic is better. Therefore, it is not surprising this is a debatable approach. Again, this was another reason why I am looking forward to see the investigators responses to  the 1st reviewer's comments.  I agree that the comparison of the methods of deriving the percentage contribution would be of interest but was not expecting that to be included.   I look forward to this comparison being published.

I confirm that I have read this submission and believe that I have an appropriate level of expertise to confirm that it is of an acceptable scientific standard.

# Response

**Published:** 2018-12-05

**Authors:** Adriani Nikolakopoulou

**Affiliations:** Institute of Social and Preventive Medicine, Switzerland

We are grateful to Dr. Annette M. O’Connor for her time and valuable comments and for recommending approval of our paper. We believe that we have been able to address all the comments raised and we have prepared a next version (Version 3) of the paper. Below are the detailed replies to reviewer’s comments; our responses to the first reviewer, Dr. Jochem König, can be found in https://f1000research.com/articles/7-610/v2.

**Referee Report: Annette M. O'Connor, Department of Veterinary Diagnostic and Production Animal Medicine, College of Veterinary Medicine, Iowa State University, Ames, IA, USA**

**Approved**

**This paper described an updated approach to deriving the percentage contributions of the direct comparison used in treatment estimates. In the interests of full disclosure, I am not a statistician and was reviewing from the view point of an applied user of NMA. There are several papers on this topic in recent years, and it is an active body of work in this area. The article is straightforward to read, although made considerably more readable if one is aware of the content in references, 3,4 and 5.**

**I have to admit that this is an example of why I appreciate the open review system. I enjoyed the paper and found it easy to read and follow, but the others reviewer's comments were a significant contribution also. I don't have any additional critique of the proposed approach, but I look forward to seeing how the investigators address those or provided some discussion, as this would help the less statistically inclined reader like me. Again, the reason I wanted this, is that I am an applied user and such discussions are very helpful. In particular, I would like to have seen some discussion of the 1st comment. I would also like to see if the comment about Figure 3 could be incorporated - perhaps not feasible.**

Thank you very much for your positive feedback. Our answers to the first reviewer, Dr. Jochem König, can be found in https://f1000research.com/articles/7-610/v2. In summary, regarding his first comment, we distinguish between representation of contribution of pieces of evidence according to a study characteristic (addressed in this paper) and quantification of the impact of within-study bias in NMA treatment effects (subject of future research). A new paragraph in the Discussion highlighting these concerns reads:

*“Study limitations may lead to biased NMA treatment effect; however, the amount and direction of bias in the NMA treatment effect as a result of the within-study bias is not straightforward to define and is not currently accommodated within the proportion contribution matrix. First, a single biased trial may affect an entire indirect route; thus, even if its proportion contribution is small, its consequences in the estimation of the NMA treatment effect may be important. Second, the direction of bias across studies involved in a stream may vary. For example, bias in two comparisons in the same stream may either cancel out or add-up in favor of one of the two treatments. We aim to extend the methods presented in this paper to develop a network meta-regression model that will use the direction and the amount of bias to determine whether and how much NMA treatment effect estimates will be biased as the result of within-study bias.”*

As we explain in detail in our response to Dr. Jochem König (comment 4), we did not amend Figure 3 to incorporate directionality.

**Concerning the approach to calculating the % contribution and how to weight the flows, I came away with the impression that the decision of equal split of the stream flow as arbitrary (perhaps not precisely the correct term) or perhaps pragmatic is better. Therefore, it is not surprising this is a debatable approach. Again, this was another reason why I am looking forward to see the investigators responses to the 1st reviewer's comments.  I agree that the comparison of the methods of deriving the percentage contribution would be of interest but was not expecting that to be included. I look forward to this comparison being published.**

We agree that equal splitting of stream flow is to some extent arbitrary but also pragmatic. We say in the Discussion:

*“An assumption that underlies our algorithm is the equal split of the stream flow to the involved comparisons. Although indirect effects are not weighted averages, we find this approximation to be a pragmatic approach that reasonably reflects the amount that each comparison contributes to network effects.”*

# Peer review: Reviewer response for version 1

**DOI:** 10.5256/f1000research.16071.r34177

**Published:** 2018-06-13

**Authors:** Jochem König

**Affiliations:** 1 Division of Paediatric Epidemiology, Institute of Medical Biostatistics, Epidemiology, and Informatics (IMBEI), Johannes Gutenberg University Medical Center, Mainz, Germany

1. The authors undertake a redefinition of the term ‘percentage contribution’ in network meta-analysis. In ordinary meta-analysis of randomized clinical trials, pooled estimates of a treatment effect can be represented as a weighted mean of treatment effects from all contributing trials. Weights are positive and sum to one and naturally constitute the ‘percentage contribution’ of each study. In network meta-analysis treatment effect estimates are defined for all pairs of treatments. Each can be represented as a two-stage estimate, firstly pooling all trials comparing the same set of treatments, respectively, which gives direct effect estimates, and then pooling direct estimates into a network based mixed treatment comparison. The linear coefficients, used in the second stage constitute the ‘contribution of direct evidence to a network based comparison’. They can be read off, from the H matrix, as described by the authors. They share some but not all aspects with weights of a two-armed meta-analysis. Aspects shared in some sense are the flow properties described by the authors and in citation [4]. The aspect not shared, is that summation to one.
  Indeed, in a purely linear graph connecting two extreme treatments through a chain of, say 10, pairwise intermediary comparisons, the network estimate is the sum of all direct effects. Any bias present in one direct effect estimate translates 1:1 into the network based estimate. Accordingly, the entries of the H matrix are all equal to 1 in the relevant row.
  Both, Salanti [3] and the present paper aim to rescale or transform the rows of the H matrix in order to achieve a ‘sum to one’ condition. In the case of the linear graph, the percentage contribution is 10% for each direct comparison. I am not convinced, that this number adequately captures the role of each direct effect estimate in this example.
  Consider now, an example where two treatments are connected through three paths with one, two, and three edges, respectively, each with flow 1/3. Then each direct comparison has the same influence on the network based treatment effect estimate: A bias of 1 unit in one direct estimate translates into a bias of 1/3 unit in the network based estimate. These proportions are conserved in Salanti’s notion of percentage contribution, but not so in the newly introduced concept. Salanti [3] attaches a percentage contribution of 1/6 to each comparison just as if we had pooled six equally precise trials comparing the same pair of treatments. But the influence of direct comparisons is different in this network and should be characterized by the number 1/3.
  That is why I have objections against Salanti’s [3] concept of percentage contribution and even more so against the newly introduced concept, which attaches unequal contribution quantities of 1/9, 1/6 and 1/3, respectively.
  The new method should not be introduced into routine of network meta-analysis. If this paper is going to be indexed, its draw backs and caveats should be very clearly set out.
2. The concept of streams is a nice one. It allows to represent a network based treatment effect as a weighted sum of a direct and indirect effect estimates each corresponding to one path from source to sink. Note however that these effects are stochastically interdependent and hence, the aggregation of streams is different from the usual process of pooling evidence.
3. To be somewhat less destructive, I propose to arrange the network graph with the source to the left and the sink to the right and all other vertices placed, such that all directed edges point from left to right. Then, due to the flow properties, any vertical cross-section gives a set of flows that sum to one. In that sense, the untransformed rows of the H matrix already contain percentage contributions. Displays used to present voter hiking can be used to represent these flows (see e.g. www.zeit.de/politik/deutschland/2017-05/waehlerwanderung-nrw-landtagswahl-cdu-spd-fdp).
4. I propose to modify Table 3 and Figure 3 in a way that the direction of influence becomes clear. Does a positive bias in one contributing direct effect translate into a positive or negative bias of the network based effect estimate? The colored fields in Figure 3 could be labelled with meaningfully ordered pairs of treatment letters.
5. Discussion “The calculation of the percentages uses the estimated variances of direct effects and thus incorporate associated uncertainty in their estimation.”
  Note however, that in a linear network each direct effect estimate contributes equally, irrespective of the size and precision of the study.
6. Discussion “Applying the algorithm to networks of interventions can be used to quantify the contribution of potential study limitations to the NMA treatment effect estimates.”
  The two stage approach discussed in this paper is not essential. Both the flows and the percentage contribution defined in the paper can be distributed to single trials according to the weights used when pooling them into direct effect estimates. Then the risk of bias of single trials can be analyzed. A display similar to Figure 3 is possible that contains separately colored fields for individual studies.
7. Discussion, 2 ^nd^ paragraph: Concerning multi-armed designs, König et al.[4] have defined the flow into and out of the vertices in a way that does not depend on the choice of reference treatment.
  Supplementary File 3:
  1st paragraph: What is the vector space of G _xy_ over R?
  2nd paragraph: ‘The number of directed paths’ obviously refers to the number of directed paths „which suffice to ‘spend’ the flow from source to sink”.
  The authors state that, if no bridges exist, this number equals to df+1. However, it depends on the flows, too. Consider a network composed of two squares connected at one vertex. It has df=2 inconsistency degrees of freedom and generally 3 paths are needed to spend the flow between the two most distant vertices. However, if all edges have equal flows, only two paths are needed. Moreover, the connecting vertex has the same effect as the bridging edges. Hence the argument has to be extended to the case of graphs that can be split by cutting through a vertex.
  Note also, that a network estimate that compares treatments from different subgroups of treatments connected through a bridge or a ‘breaking’ vertex can be written as the sum of three respective two subnetwork effect estimates: one comparing the source treatment to the bridge post, one for the bridge, if present, and one comparing the second bridge post to the sink. Because it is an unweighted sum, the contribution of the three (or two) summands should be equal.

I confirm that I have read this submission and believe that I have an appropriate level of expertise to confirm that it is of an acceptable scientific standard, however I have significant reservations, as outlined above.

# Response

**Published:** 2018-08-19

**Authors:** Adriani Nikolakopoulou

**Affiliations:** Institute of Social and Preventive Medicine, Switzerland

We are grateful to Dr. Jochem König for the time and effort he spent to review our paper. We believe that his valuable comments and suggestions have substantially improved the manuscript. We have addressed all his comments and revised the paper accordingly. Below you can find detailed replies to the reviewer’s suggestions and comments.

**Referee Report 13 Jun 2018**

**Jochem König** **, Division of Paediatric Epidemiology, Institute of Medical Biostatistics, Epidemiology, and Informatics (IMBEI), Johannes Gutenberg University Medical Center, Mainz, Germany**

**Approved with Reservations**

**1. The authors undertake a redefinition of the term ‘percentage contribution’ in network meta-analysis. In ordinary meta-analysis of randomized clinical trials, pooled estimates of a treatment effect can be represented as a weighted mean of treatment effects from all contributing trials. Weights are positive and sum to one and naturally constitute the ‘percentage contribution’ of each study. In network meta-analysis treatment effect estimates are defined for all pairs of treatments. Each can be represented as a two-stage estimate, firstly pooling all trials comparing the same set of treatments, respectively, which gives direct effect estimates, and then pooling direct estimates into a network based mixed treatment comparison. The linear coefficients, used in the second stage constitute the ‘contribution of direct evidence to a network based comparison’. They can be read off, from the H matrix, as described by the authors. They share some but not all aspects with weights of a two-armed meta-analysis. Aspects shared in some sense are the flow properties described by the authors and in citation [4]. The aspect not shared, is that summation to one.**

****

**Indeed, in a purely linear graph connecting two extreme treatments through a chain of, say 10, pairwise intermediary comparisons, the network estimate is the sum of all direct effects. Any bias present in one direct effect estimate translates 1:1 into the network based estimate. Accordingly, the entries of the H matrix are all equal to 1 in the relevant row.**

****

**Both, Salanti [3] and the present paper aim to rescale or transform the rows of the H matrix in order to achieve a ‘sum to one’ condition. In the case of the linear graph, the percentage contribution is 10% for each direct comparison. I am not convinced, that this number adequately captures the role of each direct effect estimate in this example.**

****

**Consider now, an example where two treatments are connected through three paths with one, two, and three edges, respectively, each with flow 1/3. Then each direct comparison has the same influence on the network based treatment effect estimate: A bias of 1 unit in one direct estimate translates into a bias of 1/3 unit in the network based estimate. These proportions are conserved in Salanti’s notion of percentage contribution, but not so in the newly introduced concept. Salanti [3] attaches a percentage contribution of 1/6 to each comparison just as if we had pooled six equally precise trials comparing the same pair of treatments. But the influence of direct comparisons is different in this network and should be characterized by the number 1/3.**

****

**That is why I have objections against Salanti’s [3] concept of percentage contribution and even more so against the newly introduced concept, which attaches unequal contribution quantities of 1/9, 1/6 and 1/3, respectively.**

****

**The new method should not be introduced into routine of network meta-analysis. If this paper is going to be indexed, its draw backs and caveats should be very clearly set out.**

 Thank you for this insightful comment. We agree with the point you make which we find that enlightens potential applications or extensions of the presented method to account for bias in network meta-analysis.

 We would like to make a distinction between *'representation of the contribution of pieces of evidence in a 0 to 1 scale*' and *'using this percentage contributions to represent and measure the impact and directionality of potential bias*'. In this paper, we focus on the first aspect of translating the  matrix into percentages and we subsequently apply it to depict the contribution of pieces of evidence according to a study characteristic. Our aim is to give a representation of the evidence that each comparison –or study, see our response to comment 6 below- contributes to the estimation of each NMA treatment effect without considering how this evidence could bias the results. Note that the bar plot of figure 3 gives a picture of the contribution of high, moderate or low risk of bias evidence without giving insight on the amount or direction of bias.

 Quantification of bias in the direct evidence and its impact on the NMA estimates is subject of further research but not addressed within this paper. We have now added a paragraph in the Discussion to highlight the limitations of the presented method as you recommend. The paragraph reads:

*“Study limitations may lead to biased NMA treatment effect; however, the amount and direction of bias in the NMA treatment effect as a result of the within-study bias is not straightforward to define and is not currently accommodated within the percentage contribution matrix. First, a single biased trial may affect an entire indirect route; thus, even if its percentage contribution is small, its consequences in the estimation of the NMA treatment effect may be important. Second, the direction of bias across studies involved in a stream may vary. For example, bias in two comparisons in the same stream may either cancel out or add-up in favor of one of the two treatments. We aim to extend the methods presented in this paper to develop a network meta-regression model that will use the direction and the amount of bias to determine whether and how much NMA treatment effect estimates will be biased as the result of within-study bias.”*

**2. The concept of streams is a nice one. It allows to represent a network based treatment effect as a weighted sum of a direct and indirect effect estimates each corresponding to one path from source to sink. Note however that these effects are stochastically interdependent and hence, the aggregation of streams is different from the usual process of pooling evidence.**

 Thank you for this comment; indeed the aggregation of streams is not equivalent to the aggregation of studies in a pairwise meta-analysis. We write in ‘Percentage contributions of direct comparisons’:

*“* *It can be shown that an NMA effect is a linear combination of the direct effects combined within paths.* *”*

 And we now added in the same section:

*“Equation 5 represents θ _xy_^N^* *as a weighted sum of direct and two indirect effect estimates; the effects are stochastically interdependent and, hence, their aggregation is different from the aggregation of studies in a pairwise meta-analysis.”*

**3. To be somewhat less destructive, I propose to arrange the network graph with the source to the left and the sink to the right and all other vertices placed, such that all directed edges point from left to right. Then, due to the flow properties, any vertical cross-section gives a set of flows that sum to one. In that sense, the untransformed rows of the H matrix already contain percentage contributions. Displays used to present voter hiking can be used to represent these flows (see e.g.** **www.zeit.de/politik/deutschland/2017-05/waehlerwanderung-nrw-landtagswahl-cdu-spd-fdp** **).**

 Thank you, we agree that horizontal representation is more intuitive. We re-arranged figures 1 and 2 accordingly.

**4. I propose to modify Table 3 and Figure 3 in a way that the direction of influence becomes clear. Does a positive bias in one contributing direct effect translate into a positive or negative bias of the network based effect estimate? The colored fields in Figure 3 could be labelled with meaningfully ordered pairs of treatment letters.**

 Thank you very much for this comment which we believe is related to your comment 1. We agree that such a representation would be useful but we believe it could confuse readers with respect to the scope of this paper which is not to quantify the impact of within-study bias. As said in comment 1, the scope of this paper is to represent the percentage contributions of each piece of evidence; combining this information with information of study characteristics in a figure such as figure 3 may be a useful representation of the contribution of these characteristics to the estimation of NMA treatment effects. The influence of these characteristics, though, is not straightforwardly derived and it is subject to further research. Thus, we would prefer to leave Table 3 and Figure 3 unmodified and construct such representations of the direction of bias in future work where we aim to develop models to account for study-level bias using the presented methodology.

 Please also see the reply to comment 1 and the addition to the Discussion.

**5. Discussion “The calculation of the percentages uses the estimated variances of direct effects and thus incorporate associated uncertainty in their estimation.”**

**Note however, that in a linear network each direct effect estimate contributes equally, irrespective of the size and precision of the study.**

 We agree this statement is confusing and hence deleted this phrase from the Discussion. The paragraph now reads:

*“In this paper, we present a new approach to derive percentage contributions of individual studies to the treatment effect estimates in NMA. We made use of the fact that the composition of network treatment effect estimates can be interpreted as a flow of evidence. An assumption that underlies our algorithm is the equal split of the stream flow to the involved comparisons. Although indirect effects are not weighted averages, we find this approximation to be a pragmatic approach that reasonably reflects the amount that each comparison contributes to network effects.”*

**6. Discussion “Applying the algorithm to networks of interventions can be used to quantify the contribution of potential study limitations to the NMA treatment effect estimates.”**

**The two stage approach discussed in this paper is not essential. Both the flows and the percentage contribution defined in the paper can be distributed to single trials according to the weights used when pooling them into direct effect estimates. Then the risk of bias of single trials can be analyzed. A display similar to Figure 3 is possible that contains separately colored fields for individual studies.**

 Thank you for this comment; we agree that such a distribution of percentage contributions to single trials is possible and relevant and we have updated the paper accordingly.

 1.We added an extra paragraph/subsection *‘Percentage study contributions’* where we elaborate on the process you describe. The new paragraph reads:

*“* *Matrix **P*** *(Table 3) shows the percentage contributions of each direct comparison to each NMA treatment effect estimate. These percentages can be distributed to individual studies within each comparison according to their weights from direct meta-analyses. For example, p _xy_=63.5%* *and there are two studies examining the xy* comparison. The individual study weights for the two studies are 0.69 and 1.54 resulting to study percentage contributions of  (0.69 /(0.69+1.54))*63.5%=19.6% and (1.54/(0.69+1.54))*63.5%=43.8% to the xy *NMA treatment effect estimate. The application of this process to the entire matrix **P*** leads to the matrix P ^*^ *shown in Table 4.* *Adjusted weights* *as proposed by* *Rücker* *& Schwarzer* *(9)* *are used for* *multi-arm studies.”*

 2.Section *‘Using percentage contributions to quantify the impact of a characteristic in a direct comparison’ *has been renamed to *‘Using percentage study contributions to quantify the impact of a characteristic in a direct comparison’ *and follows the new ‘Percentage study contributions’ section. Text has been updated to refer to studies instead of direct comparisons and Figure 3 (and the new Table 4) now displays percentage study contributions to NMA treatment effect estimates.

 3.The CINeMA (Confidence In Network Meta-Analysis) http://cinema.ispm.ch/ software has also been updated to display the bar chart using study contributions.

 4. The first sentence of the Discussion now reads:

*“In this paper, we present a new approach to derive percentage contributions of individual studies to the treatment effect estimates in NMA.”*

**7. Discussion, 2 ^nd^ paragraph: Concerning multi-armed designs, König et al.[4] have defined the flow into and out of the vertices in a way that does not depend on the choice of reference treatment.** 

 Thank you for this comment, we could indeed use the **H** matrix as described in section 3.3.1 of König et al.[4] (and implemented in netmeta as H.tilde) for multi-arm designs; some concerns about the interpretability of such a matrix have previously precluded us of doing so. In fact, the algorithm could be applied to any **H** matrix as long as its rows have the flow properties described in König et al.[4] and in our section ‘Comparison graph’. We have removed the discussion on multi-arm trials from the Discussion and added the following paragraph at the end of the Methods section:

*“The starting point for the developed algorithm was a simplified version of the* **H** *matrix* *that does not consider the correlation induced by multi-arm trials. Alternatively, one could use the* *matrix* *as described by König et al.* *(4)* *that properly accommodates multi-arm designs. Note that any matrix whose rows can be interpreted as flow networks can be used as the starting point of the algorithm.”*

**Supplementary File 3:**

**1st paragraph: What is the vector space of G _xy_ over R?**

 Thank you for this comment; as it was not clear and in order to simplify this part, we eliminated the notions of ‘vector space’ and ‘basis’ in the document. It now reads:

 “However, in the general case, the number of paths used in the algorithm is smaller than the number of elements of Π. The number of directed paths which suffice to ‘spend’ the flow from source to sink is less or equal to df+1 where df is the inconsistency degrees of freedom in the Lu and Ades inconsistency model (1), df=D-T+1.”

**2nd paragraph: ‘The number of directed paths’ obviously refers to the number of directed paths „which suffice to ‘spend’ the flow from source to sink”.**

 Thank you, we corrected it. 

**The authors state that, if no bridges exist, this number equals to df+1. However, it depends on the flows, too. Consider a network composed of two squares connected at one vertex. It has df=2 inconsistency degrees of freedom and generally 3 paths are needed to spend the flow between the two most distant vertices. However, if all edges have equal flows, only two paths are needed. Moreover, the connecting vertex has the same effect as the bridging edges. Hence the argument has to be extended to the case of graphs that can be split by cutting through a vertex.**

**Note also, that a network estimate that compares treatments from different subgroups of treatments connected through a bridge or a ‘breaking’ vertex can be written as the sum of three respective two subnetwork effect estimates: one comparing the source treatment to the bridge post, one for the bridge, if present, and one comparing the second bridge post to the sink. Because it is an unweighted sum, the contribution of the three (or two) summands should be equal.**

 Thank you very much for these interesting notes with which we totally agree. We extended the ‘Number of directed paths’ section in Supplementary File 3 to include discussion on the ‘breaking’ vertex situation and the consequences you describe. In particular we wrote:

*“Another situation where the number of directed paths which suffice to ‘spend’ the flow from source to sink is less than df+1* *elements* *occurs when a vertex has at least two inflows and two outflows, all equal between them. In such a situation the flow from source to sink may be spent in less than df+1* *directed paths. Such a vertex will be called breaking vertex. Note that an NMA treatment effect estimate for treatments separated by a bridge or a breaking vertex (say treatments x* *and y* *) can be seen as an “indirect” comparison through either the intermediate bridge (constituting by treatments b _1_ and b2) or the intermediate breaking vertex (b0)* *.*

*In particular, in the case of a bridge, the NMA treatment effect estimate is derived as*

*θ _xy_^N^=θ _xb1_^N^+θ _b1b0_^N^+θ _b2y_^N^*

*and in the case of a breaking vertex the NMA treatment effect estimate is*

*θ _xy_^N^=θ _xb0_^N^+θ _b0y_^N^*

*It is, thus, implied that in such a case the separate subnetworks contribute equally to the estimation of θ _xy_^N^.”*

- **Is the rationale for developing the new method (or application) clearly explained?**

**Yes**

- **Is the description of the method technically sound?**

**Yes**

- **Are sufficient details provided to allow replication of the method development and its use by others?**

**Yes**

- **If any results are presented, are all the source data underlying the results available to ensure full reproducibility?**

**Yes**

- **Are the conclusions about the method and its performance adequately supported by the findings presented in the article?**

**Partly**

**Competing Interests:** **No competing interests were disclosed.**

**I have read this submission. I believe that I have an appropriate level of expertise to confirm that it is of an acceptable scientific standard, however I have significant reservations, as outlined above.**
